# Decellularized Porcine Tendon Xenografts Provide a Viable Option in Anterior Cruciate Ligament Reconstruction

**DOI:** 10.7759/cureus.109585

**Published:** 2026-05-25

**Authors:** Stefano Petrillo, Kristian Samuelsson, Eric H Senorski, Sergio Romagnoli, Matteo Marullo

**Affiliations:** 1 Prosthetic Surgery Centre, IRCCS Ospedale Galeazzi-Sant'Ambrogio, Milan, ITA; 2 Orthopaedics and Rehabilitation Department, University of Gothenburg, Gothenburg, SWE; 3 Health and Rehabilitation Department, University of Gothenburg, Gothenburg, SWE

**Keywords:** acl, anterior cruciate ligament, porcine, reconstruction, xenograft

## Abstract

Background: The anterior cruciate ligament (ACL) is one of the most commonly injured ligaments, and ACL reconstruction (ACLR) is projected to steadily increase. While autografts are the gold standard, the limited harvest volume and donor site morbidity have spurred the search for viable alternatives. A decellularized porcine xenograft is currently available; therefore, we have investigated patient outcomes in a series of patients who have received this graft for ACLR.

Methods: Patients were eligible for inclusion in this study if they had undergone ACLR, either primary or revision, and had received a decellularized tendon xenograft (Orthopure XT, Tissue Regenix, Leeds, UK) as the ACL graft. All patients underwent surgery by the same surgeon at the same hospital (IRCCS Ospedale Galeazzi, Milan, Italy) and followed standardized care for ACLR, including surgery and post-operative rehabilitation. The outcome data collected were the International Knee Documentation Committee (IKDC) score, Tegner-Lysholm, and visual analog scale (VAS) for pain. Outcome data were analysed using a repeated measures ANOVA, with α=0.05.

Results: Of the 29 patients who underwent ACLR, 24 (83%) were male. The mean age at surgery for male patients was 43.6 ± 9.8 years, while for women, it was 40.8 ± 15.1 years. The mean BMI was 25.8 ± 3.4 for men and 25.4 ± 2.9 for women. Follow-up was collected at a mean of 27.4 ± 9.6 months after surgery. The mean post-operative IKDC score was 94.9 ± 2.6 (95% CI: 93.9-95.9). The Tegner-Lysholm score improved from a pre-operative mean of 43.3 ± 5.7 (95% CI: 41.1-45.4) to a post-operative mean of 92.7 ± 3.6 (95% CI: 91.3-94; p<0.001). The VAS pain improved from a mean of 3.0 ± 1.4 to a post-operative mean of 0.4 ± 0.7 (p < 0.001). During the follow-up period, there was one traumatic failure (3%) and one acute infection (3%). The positive responder rate was 97%.

Conclusion: The short-term follow-up outcomes in this small, retrospective cohort indicate that this decellularized xenogeneic graft, processed from porcine tendon, can provide a viable option in ACL reconstruction.

## Introduction

Although anterior cruciate ligament (ACL) reconstruction (ACLR) has become a common surgery, there is no universally agreed-upon graft choice for this procedure, which has left the choice of graft as an area of debate for several years [1]. While autograft is considered the gold standard for ACLR, its disadvantages include requiring an additional surgical site for tissue harvest, resulting in donor site morbidity [2]. The use of allografts in ACLR dates to 1988 [3], and this procedure has demonstrated outcomes, as evidenced by improved patient-reported outcome scores [4]. However, it should be noted that patients with lower activity levels and older age have been described as the best candidates to receive an allograft [5]. Allografts may offer an initial mechanical strength that is similar to autografts, but there is an important distinction in that allografts have no donor site morbidity for the patient [6]. Importantly, allografts have been reported with a higher incidence of graft failure after ACLR, compared with autografts [7]. There are also limited sources of allografts from young, healthy donors, and the ones available are expensive [8]. Other downsides to allograft use are the perceived risk of infection (although undocumented) and the need for deep-frozen storage, which requires thawing prior to surgery [9].

Such concerns with allografts and synthetic grafts in ACLR have led to the development of xenografts, which are tissues of animal origin [10]. As it pertains to the ACL, xenografts have been reported to possess the appropriate graft dimensions and tissue strength to support their use in ACLR, but early studies reported a high failure rate [11]. Recent advances in the processing of xenograft tissue, which removes the α-Gal epitopes as well as viable cells, can prevent or reduce the immune response [12,13]. In orthopaedics, a porcine scaffold has seen extensive use in chondral repair, with over 20 years of clinical use [14,15]. Specific to ACLR and porcine-derived xenografts, Butler et al. reported that treated xenografts may be a viable option for ACL repair [16], with longer-term data from a rather small cohort having been recently published [17]. However, the xenograft used in the studies by Butler et al. [16,17] is no longer available.

As a result of the need for alternative transplants for ACLR, a xenograft has been developed that is derived from porcine superflexor tendons (OrthoPure XT; Tissue Regenix, Leeds, UK). The tendon was chosen as it has the appropriate mechanical strength and physical dimensions for ACLR [18]. Following processing for decellularization, the ultimate tensile strength (UTS) of the xenograft was measured to 52.5 MPa [18], which compares well with the UTS of native human ACL of 38 MPa [19]. Although these mechanical tests imply a useful role for such xenografts in human use, rates of failure and infection have been documented in previous studies in which xenogeneic grafts have been used in ACLR [17,20].

Importantly, there is a paucity of clinical data related to the use of these xenografts in ACLR. One study has recently been published and reported an improvement in the International Knee Documentation Committee (IKDC), Lysholm scores, as well as improved Lachman and pivot-shift tests during 60 months of follow-up [21]. While the outcomes were generally positive, only 48% of the patients were available at the final follow-up.

Therefore, there is a clear need to document outcomes for the safety and performance of grafts for ACL reconstruction, especially in real-world situations. In order to assess the utility of a new xenograft, we aimed to test the hypothesis that a decellularized, xenogeneic graft would provide an improvement in functional scores and a reduction in pain, at a minimum 20-month follow-up, following primary or revision reconstruction of the ACL.

## Materials and methods

Study design

This was a retrospective cohort study that included patients undergoing primary ACL reconstruction (ACLR) at one site. Documentation of patient information and outcomes was made on electronic case report forms. Ethical approval was obtained from the ethics review board of San Raffaele University of Milan, Italy (ALL-ACL study).

Study participants

The participants in this study were a series of 29 consecutive patients who had undergone ACLR using a xenograft of porcine tendon. The inclusion criteria were the implantation of the xenograft as part of the ACLR and consent to have their data used in the study. The exclusion criterion was a concomitant unilateral knee arthroplasty (UKA). As this was a consecutive case series, the final inclusion criterion was consent to have their data used in the study.

Xenograft

**Figure 1:**
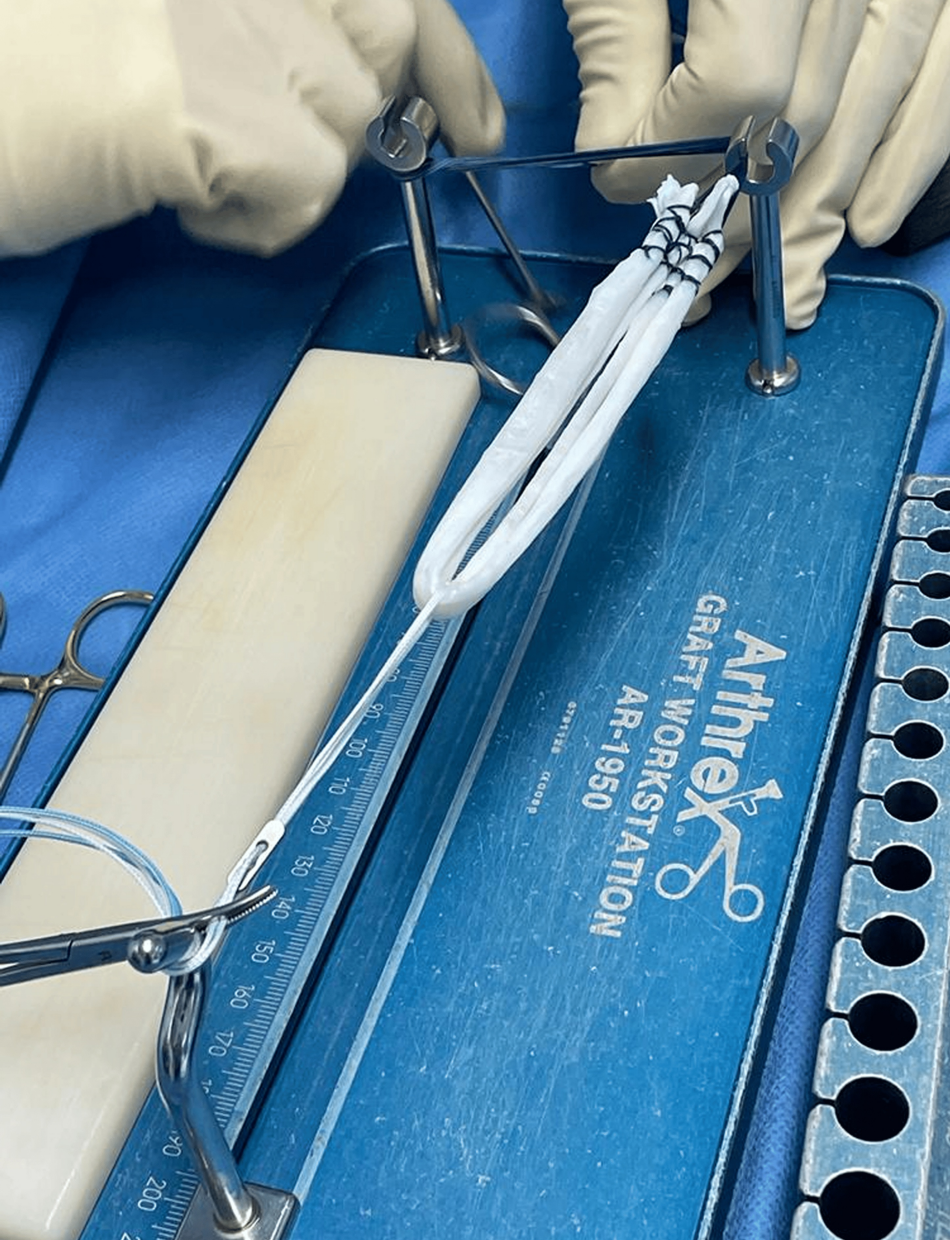
The porcine xenograft used for ACL reconstruction. ACL: anterior cruciate ligament

The xenograft that we implant is the Orthopure XT (Tissue Regenix Group, Leeds, UK), which is a decellularized porcine xenograft, as shown in Figure 1. In this process, a graft from a porcine digital extensor tendon (pDET) of the hind limb is processed via treatment at freezing temperatures, exposure to salt and nuclease solutions, and processing with a mild detergent to remove all viable cells and native components that have the potential to elicit an immune response [22,23]. Following this, the graft is suspended in normal saline (0.9%) in a blister package, and then it undergoes terminal sterilisation with 25 kGy gamma irradiation, which permits the graft to be stored at room temperature.

Surgical technique

All patients underwent an anteromedial technique using a porcine xenograft, as shown in Figure 1 (OrthoPure XT, Tissue Regenix, Leeds, UK), which was used for the reconstruction. The patient, under spinal anaesthesia, was placed in the supine position. The involved inferior limb was in a leg holder permitting knee movement from 0° to 110°. The surgical procedure started with a diagnostic arthroscopy to evaluate the ACL rupture and to treat any meniscal lesions. Then, using a calibrated tibial guide, a bone tunnel was created at the level of the remnant fibres of the injured ACL. The femoral tunnel was created through the anteromedial portal at the level of the ACL femoral footprint. Finally, the porcine graft was passed through the bone tunnels and fixed proximally with an adjustable cortical button in the femur and with a bioscrew in the tibia.

Rehabilitation

Rehabilitation followed standard guidelines. In week one, a progressive regimen was started, which included passive motion and isometric exercises with a goal to achieve 120^o^ range of motion, as well as full mobility with the appropriate use of walking aids. In weeks two through six, strengthening and range of motion exercises were continued, as well as the addition of proprioceptive exercises, with the goal being a full range of motion with no lag. From week 6 through 12, exercises progressed as appropriate with the addition of core stability to the strengthening, as the goal was to achieve a normal gait pattern with no anterior knee pain. Finally, from months three through six, patients progressed to full mobility and function, with the addition of plyometrics and jogging, and progression to running as normal gait was reestablished, but no contact sports.

Study outcomes

The primary outcome was the change in the Tegner-Lysholm score between pre-operative and the final follow-up visit [24]. At the same time, an assessment of pain was done using the visual analog scale for pain (VAS) [25]. The International Knee Documentation Committee (IKDC) was completed at the six-month follow-up [26]. Because the data from this study followed the standard of care, there were no additional, predefined follow-up visits.

Statistical analysis

The pre-operative demographic data were compared between patient sexes using a Mann-Whitney U-test, due to the small number of female patients. The assessors were not blinded. For the IKDC, the mean and standard deviation were calculated. The pre- and post-operative Tegner-Lysholm scores were assessed using repeated measures ANOVA. The change in the Tegner-Lysholm scores was further evaluated in relation to age at surgery and BMI. Statistical analyses were performed using an online statistical calculator (numiqo, which has no version number) [25], and the alpha was set to 0.05. There was no plan for missing data, as we only included patients who had pre-operative and post-operative assessments. The rate for positive response was determined by an IKDC score greater than 76 and the lack of failure or revision surgery during the follow-up.

## Results

Patients

The demographic data for the 29 patients in the cohort are presented in Table 1. Of these, 24 (83%) cases were primary reconstructions, and five (21%) were revisions, although all cases among women were primary reconstructions (100%). It should be noted that the p-values in Table 1 reflect the results of a Mann-Whitney test, but the number of meniscectomy patients in the female subset precluded a meaningful comparison of the distribution of cases concerning meniscectomy.

**Table 1:** Demographic characteristics of the patients.

Variables	Male	Female	p
n	24 (83%)	5 (17%)	-
Primary	19 (79%)	5 (100%)	-
BMI	25.8 ± 3.4	25.4 ± 2.9	0.89
Age at Surgery, Years	43.6 ± 9.8	40.8 ± 15.1	0.77
Time in Surgery, Minutes	34.5 ± 11	41 ± 15.5	0.53
Pre-operative Tegner-Lysholm	43.8 ± 5.9	40.6 ± 3.8	0.32
Concomitant			
Partial Medial Meniscectomy	12	2	na
Partial Lateral Meniscectomy	2	2	na

Tegner-Lysholm scores

**Figure 2:**
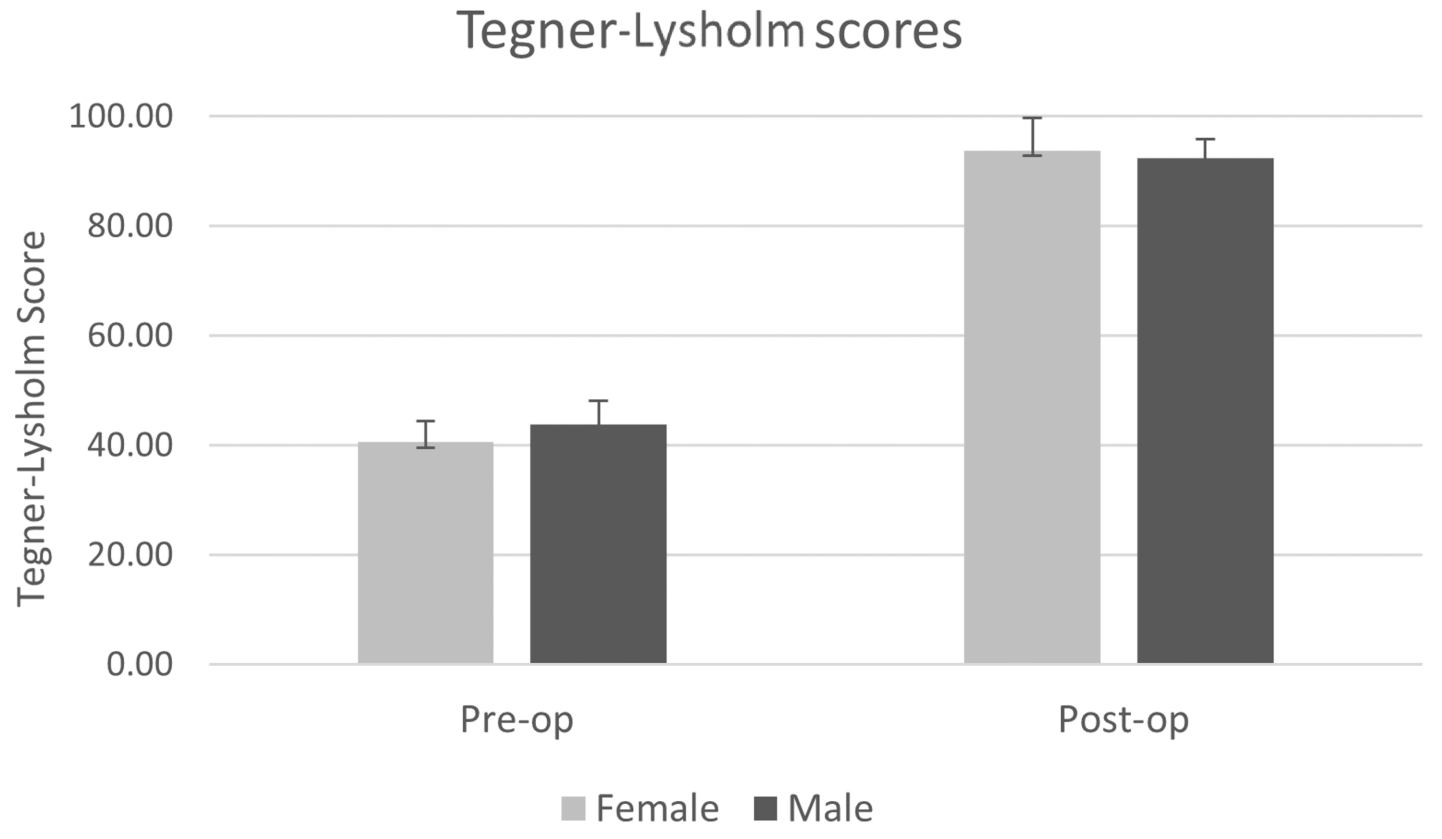
The Tegner-Lysholm scores from pre-operative to last follow-up.

The Tegner-Lysholm scores showed a significant difference between pre-op and post-op scores (p < 0.001). The pre-op scores for the entire cohort were measured at 43.3 ± 5.7 (95% CI: 41.1-45.4), while the post-operative Tegner-Lysholm scores improved to 92.7 ± 3.6 (95% CI: 91.3-94). There was no difference in outcomes with regard to partial meniscectomy (p = 0.7). This is depicted in Figure 2.

Visual analog scale (VAS)

**Figure 3:**
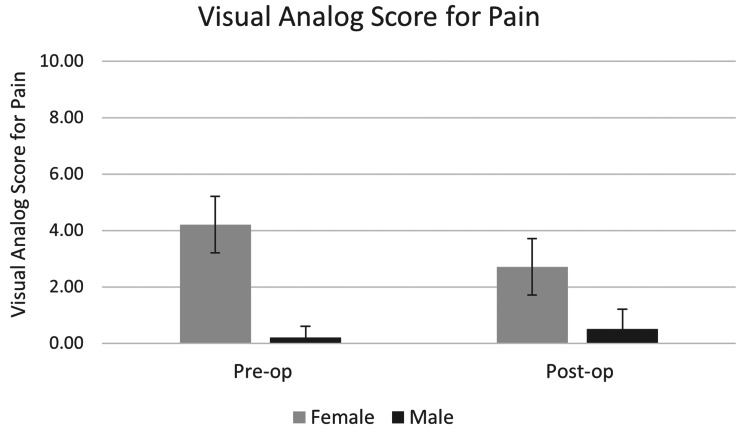
The change in VAS for pain from pre-operative to last follow-up. VAS: visual analog scale

The VAS for pain showed a significant change from the pre-operative value of 3.0 ± 1.4 to a post-operative mean of 0.4 ± 0.7 at the date of the last follow-up (p < 0.001), which is depicted in Figure 3. There was no difference in the VAS was compared between men and women (p = 0.143). There was no difference in outcomes with regard to meniscectomy (p = 0.752). We had grouped all meniscectomies, as there were only three lateral and one bilateral meniscectomy.

International Knee Documentation Committee (IKDC)

The post-operative IKDC score for the entire cohort was 94.9 ± 2.6. There was no difference in the results when comparing revision ACLR (95 ± 2.2) and primary ACLR (94.8 ± 2.7). A correlation analysis revealed a weak trend towards a relationship between the outcome score and the patient's age at the time of surgery (p = 0.249, r = 0.2). There was no relationship between BMI and IKDC score (p = 0.65, r = 0.1). Whether or not the patient had a meniscectomy had no effect on the final IKDC score (p = 0.278)

Associations between Tegner-Lysholm and demographic data

**Figure 4:**
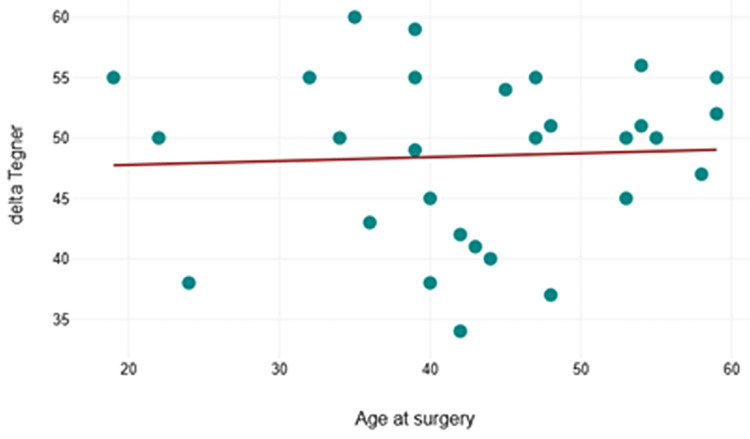
The change in the Tegner-Lysholm score in relation to the patient's age at surgery.

There were no relationships noted between the change in Tegner-Lysholm score and either BMI (p = 0.708) or the age at the time of surgery (p = 0.802). The relationship between age and the improvement in the score is shown in Figure 4. Concerning the patient's sex, the small number of female patients (n=5, 17%) precluded a meaningful statistical comparison of the effect of the patient's sex on their outcomes.

Safety

Over the course of the follow-up period, there was one failure, one acute infection, and one report of post-operative cyst formation. The acute infection was a *Staphylococcus aureus *infection, which is one of the most common pathogens after ACL reconstruction [26]. This infection was managed with arthroscopic lavage and antibiotics, and it resolved after 10 days. The sole case of graft failure was a reinjury that happened nine months after surgery, while the patient had been playing football. 

Positive responders

An IKDC score of 76 points was considered to meet the criteria for the patient acceptable symptom state (PASS) and was therefore considered to be a positive response to the surgery [27]. Considering the one failure, the positive responder rate was 97%.

Imaging

**Figure 5:**
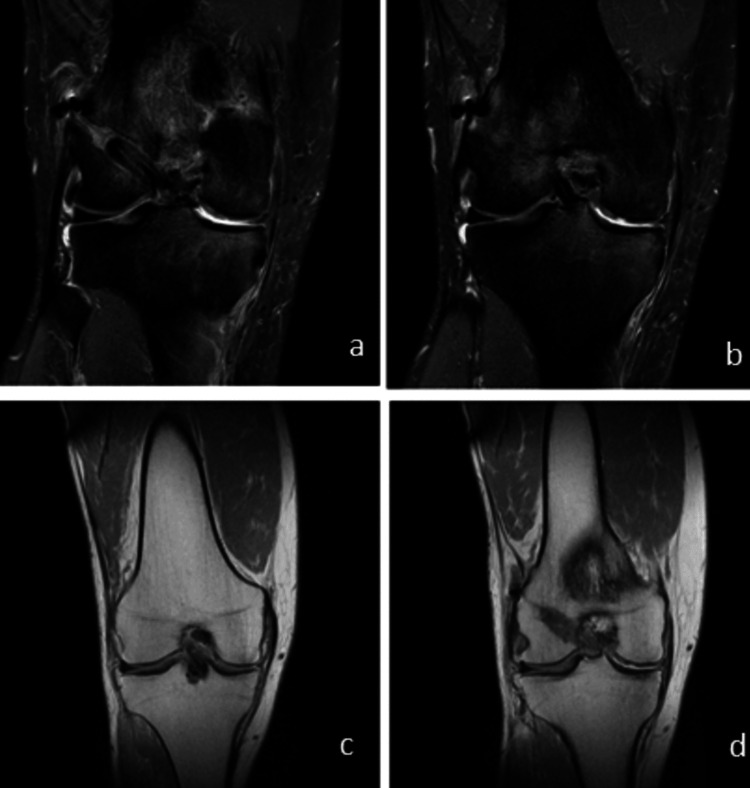
A T2-weighted image of a patient at one-year follow-up. MRI coronal image showing integration of the graft in the femoral tunnel (a) and healthy ligament (b). MRI coronal image showing healthy ACL (c) and graft integration (d).

Specific to the remodelling of this graft tissue after implantation, Figure 5 shows the MRI at one-year follow-up in a 52-year-old male patient. The patients showed a Howell grade 1 graft, with a signal similar to a quadriceps tendon or PCL. Thus, the radiological evidence indicates that religamentization is well advanced in this case. 

## Discussion

The main findings of this retrospective, single-centre cohort study in which a porcine xenograft was used in ACLR were that the patients demonstrated a significant improvement in the Tegner-Lysholm score, along with a significant reduction in the VAS score for pain. The values for Tegner-Lysholm exceeded the minimum clinically important difference (MCID) for ACLR, while the IKDC, even though there was only one post-operative measure, exceeded a generally accepted score for PASS (76 points). When this is viewed alongside the lack of significant post-operative complications and the absence of failures, it is apparent that this decellularized porcine tendon xenograft offers a safe and effective alternative to autografts in ACL reconstruction surgery.

While allografts have been used for many years, the failure rate has been reported to be notably higher than seen with autograft ACLR [27,28]. Considering this, xenografts have been viewed as a potential source of graft material. While an initial study using a porcine BTB graft had shown promising results [13], the graft that had been used in that study is no longer marketed. With respect to long-term data, a 20-year follow-up in a small number of patients (n = 5, of 10 initially treated) reported that those patients had a stable knee with no degradation in patient-reported outcome measures (PROMs), physical exam, or appearance [14].

In contrast to this, the results of a randomised clinical trial noted that, although pain and function were comparable between groups, a high infection rate (20.6%) was reported in patients who had received a porcine BTB, although the specific graft was not identified [17]. The authors in that paper had reported that the xenografts had been terminally sterilized with irradiation, which one would expect to impact failures, but a higher rate of infections was the cause of most failures in the xenograft group, which is why antibiotic prophylaxis is commonly administered for ACL surgery [29].

The clinical suitability of the porcine xenograft that we used had been demonstrated in a recent study, which published five-year follow-up data, showing improvements in clinical outcome scores, along with no evidence of an immunogenic response [18]. The IKDC scores that we recorded are similar to the scores that Hunt et al. had presented for both 24 and 60 months of follow-up [18]. Similarly, the patients in our cohort also showed no immunogenic response. These positive outcomes lend further support to the use of this porcine xenograft in ACL reconstruction. While an off-the-shelf xenograft may likely never be a complete substitute for an autograft, they merit consideration in comparison to allografts.

To date, we have seen only one post-operative infection and one post-operative cyst formation, which indicates that the graft has been effectively processed to minimize these risks. The infection was a *S. aureus *infection, which is one of the most common post-operative infections following ACLR [25]. The sole failure was a reinjury that happened while the patient had been playing football, which contradicts one of our requirements for patient selection for this graft (i.e., being low physical demand).

One of the advantages that we have noted is the relatively brief time in surgery. Our mean surgery time was 36 ± 12 minutes for ACLR. For isolated ACLR, it was 30 minutes, while it was slightly longer for patients undergoing partial lateral meniscectomy (40 minutes) or partial medial meniscectomy (39 minutes). Aside from the short surgery time, the lack of donor site morbidity likely means less pain for the patient, an aspect of knee surgery that has already been noted, as autografts result in more scarring than has been seen with allografts [8].

Patient selection, however, is an important aspect in treatment planning and thus obtaining optimal clinical outcomes. Patients over the age of 40 years is not an absolute value but offers a point of reference in deciding on the use of this xenograft. In our clinic, this xenograft will be the first choice for older ACLR patients, while for younger patients, we also consider their physical activity, especially regarding sports. Revision of a failed ACLR would also lend weight to the use of this xenograft, but that remains to be evaluated in further research. Overall, the individualization of treatment planning is essential in achieving optimal outcomes for the patient.

Our study is not, however, without limitations. As this was a consecutive case series of patients, the follow-up times varied, from a low of 20 months to a maximum of 44 months. Although we followed the standard of care regarding follow-up visits, a more controlled, prospective study should have standardized follow-up times. Another limitation is the lack of a pre-operative IKDC score. Although our clinic regularly collects the Tegner-Lysholm score at a pre-operative visit, this PROM is not as widely used as the IKDC; thus, a more familiar PROM would be useful when comparing study outcomes. Finally, a more standardized collection of post-operative complications and adverse events would contribute to a better understanding of the patients' reactions to this xenograft. Although the human response to xenografts has been well-documented in the past, and the processing methods of these xenografts seem to have solved the problems with host immune responses, a more detailed, systematic documentation would help better evaluate the potential for these grafts to be a reliable option in ACLR.

## Conclusions

The positive outcomes that we have noted indicate that this decellularized porcine tendon provides a safe and effective option in ACL reconstruction. The high IKDC scores and the large improvements in Tegner-Lysholm scores, in tandem with the safety data, support the use of these grafts in ACL reconstruction. However, longer-term follow-up and comparative studies are essential, as the data that we have presented are preliminary results. Furthermore, our outcomes may reflect patient selection, for example, low physical demand and older patients. Therefore, the indications for each type of graft need to be further elucidated in order to afford surgeons the best chance of providing optimal patient outcomes.
